# Mixing planting with native tree species reshapes soil fungal community diversity and structure in multi-generational eucalypt plantations in southern China

**DOI:** 10.3389/fmicb.2023.1132875

**Published:** 2023-02-22

**Authors:** Chao Li, Yuxing Xu, Zhichao Wang, Wankuan Zhu, Apeng Du

**Affiliations:** Research Institute of Fast-growing Trees, Chinese Academy of Forestry, Zhanjiang, Guangdong, China

**Keywords:** continuous planting pattern, mixed planting patterns, co-occurrence network, ITS gene sequencing, soil fungal diversity

## Abstract

The continuous planting pattern of eucalypt plantations negatively affects soil quality. A mixed planting pattern using native species implanted in pure plantations has been considered a preferable measure for this problem. However, the impact of this approachon the structure and function of fungal communities is not clear. Here, harvesting sites that had undergone two generations of eucalypt plantations were selected to investigate soil fungal community structure and the co-occurrence network characteristics in response to two silvicultural patterns involving the third generation of eucalypt plantations (E) and mixed plantations of *Eucalyptus. urograndis* × *Cinnamomum. camphora* (EC) and *E. urograndis* × *Castanopsis. hystrix* (EH). Compared with the first generation of eucalypt plantations (CK), E markedly weakened enzyme activities associated with carbon-, nitrogen-. and phosphorus-cycling. Reduced soil fungal alpha diversity, and elevated the relative abundance of Basidiomycota while decreasing the abundance of Ascomycota. In contrast, EC and EH not only enhanced fungal alpha diversity, but also reshaped fungal composition. At the class level, E caused an enrichment of oligotrophic Agaricomycetes fungi, classified into symbiotroph guild, while EC markedly decreased the abundance of those fungi and increased the abundances of Sordariomycetes, Dothideomycetes, Eurotiomycetes, and Tremellomycetes fungi, which were classified into saprotroph or pathotroph guild. Moreover, fungal network complexity and robustness topological attributes were higher or significantly higher in mixed plantations soils compared with those of pure eucalypt plantation E. Furthermore, fungal diversity, structure, and functional taxa were significantly affected by soil organic matter, pH, total nitrogen, and nitrate nitrogen.

## Introduction

In recent decades, monocultures of fast-growing plantation species have increasingly replaced temperate and tropical native forests for greater economic efficiency and to satisfy the growing demand for wood products (Teixeira et al., 2017). China has the largest area of planted forests in the world, with over 80 million hectares, accounting for 36.45% of the country’s total forest area. *Eucalyptus*, an important timber species in South China, occupied an area of 5.46 million hectares in China in 2018, accounting for 21.86% of the total area of eucalypt plantations worldwide (National Forestry and Grassland Administration, 2019). Eucalypt plantations are usually planted with single species patterns in short rotation periods (Chen et al., 2011). However, with successive planting generations, this single silvicultural model negatively affects the sustainability of soil fertility and nutrient cycling (Teixeira et al., 2017; Wu et al., 2019; Xu et al., 2020). In recent years, mixed planting of native species with eucalypts has been considered a preferred solution for this problem (Xu et al., 2019, 2021b). Soil microorganisms are essential for maintaining soil function, and the evolution of their community structure is driven by anthropogenic disturbances and soil nutrient availability, while also strongly influencing soil nutrient cycling processes (Cai et al., 2010; Chen et al., 2019). Although the eucalypt–native species mixed model may efficiently enhance soil fertility in degraded woodlands, it is still unclear how the microbial community structure and function, especially fungi, respond to changes in soil nutrient availability and decomposition substrate quality caused by native species implantation.

Soil microorganisms, as core drivers of soil quality improvement, participate in processes such as soil organic matter (OM) decomposition and nutrient cycling in forest ecosystems, and are instrumental in ecosystem regulation of soil biogeochemical processes (Geisseler and Horwath, 2009). With the transition from pure to mixed plantations, microorganisms gradually shaped new community diversity, composition, and functional characteristics in response to the changing interspecific relationships, litter quality, and soil physicochemical properties (Zhang et al., 2018; Qu et al., 2022). Studies have shown that the implantation of native species such as *Manglietia glauca*, *Castanopsis hystrix*, *Acacia mangium*, and *Cinnamomum camphora* in eucalypt plantations significantly increase the soil bacterial diversity and relative abundance of copiotrophic and nitrogen cycle-associated bacteria (Xu et al., 2021b, 2022). However, there are few studies addressing the effects of pure plantation transformation on soil fungal communities (Rachid et al., 2013; Wu et al., 2019; Liu et al., 2022). Consequently, this limits a more comprehensive assessment of soil microbial communities in response to the shifts in plantation management patterns. As an important component of soil microorganisms, fungi determine numerous ecosystem processes, participate in plant–soil energy flow and OM transformation, and possess a wide range of enzymatic capabilities involved in carbon (C), nitrogen (N), and phosphorus (P) degradation (Benoit et al., 2015; Treseder and Lennon, 2015; Uroz et al., 2016). Many fungi survive in forest soils in saprophytic or symbiotic forms and even outnumber the bacteria involved in decomposition, especially in the shallow layers of acidic soils (Maraun and Scheu, 1996). Thus, during forest conversion, fungal communities may play a more crucial role in above-and below-ground connectivity and in C-, N-, and P-cycling compared with bacterial communities (Jin et al., 2019).

Currently, soil fungi can be generally classified into symbiotic fungi, saprophytic fungi, and pathogenic fungi according to their nutritional and ecological functions (Nguyen et al., 2016). Symbiotic fungi predominantly comprise ectomycorrhizal (ECM) fungi and arbuscular mycorrhizal (AM) fungi, which improve the nutrient status of plants by establishing a reciprocal symbiosis with plants while obtaining fixed carbon from plants to complete their life cycle (Lei et al., 2022). Most AM fungi can only utilize inorganic nitrogen sources, and the depletion of such nutrients is unfavorable to AM fungal reproduction. However, ECM fungi can transfer N sources (e.g., chitin and protein) from OM, which is an important process in the mitigation of drought and nutrient stress (Read and Perez-Moreno, 2003). Eucalypt species have a dual mycorrhizal symbiosis mode (ECM and AM), and changes in soil nutrients and hosts as a result of plantation transformation may upset the balance between symbiotic fungi and hosts (Adjoud-Sadadou and Halli-Hargas, 2017). Soil saprophytic fungi are preferred to decompose recalcitrant plant residues and soil OM, with a significantly positive correlation between their relative abundance and soil fertility (Kyaschenko et al., 2017; Castano et al., 2019). Therefore, given the important role of different fungal functional guilds in plantation ecosystems, it is important to understand the response of fungal community structure to changes in plantation management patterns.

In this study, harvested sites that had undergone two generations of eucalypt plantation were selected to investigate the characteristics of soil fertility quality and fungal communities, and their coupling relationships, in response to different plantation cultivation patterns (pure and mixed plantations). The objectives of the study were: (1) elucidate the response of soil nutrient status and soil enzyme activities on the transformation of pure plantations into mixed plantations, (2) investigate the soil fungal community diversity characteristics, structural composition, and functional changes in response to the transformation, and (3) couple soil nutrient and enzyme activities with fungal diversity and community structure characteristics, and reveal the key soil environmental factors significant correlated with fungal community.

## Materials and methods

### Study area

The experimental site was located in Luogangling Forest Park, Zhanjiang, Guangdong (21°16′ N, 110°05′E), which belongs to the northern edge of the Leiqong area with a minimum elevation of 80 m and a maximum elevation of 221 m. The site is in the tropical northern humid zone, with an average annual temperature of 23.1°C and a long-term average annual rainfall of over 1,300 mm.

The study area was laid out in Guangdong Zhanjiang Eucalyptus Plantation Ecosystem Research Station in Luogangling Forest Park, on a gentle slope of low hills at 80–100 m above sea level. The soil is classified as Rhodi-Udic Ferralosols according to the Chinese Soil Taxonomy Classification (Gong, 1999), which is consistent with the Rhodic Paleudult according to American Soil Taxonomy (Staff, 2010). The experiment was conducted on harvested sites that had undergone two successive generations of eucalypt plantations (8 and 5 years of plantation cultivation, respectively) in the same area, so it can be assumed that eucalypt plantations and mixed plantations had similar geospatial heterogeneity before afforestation. Information on the plantations from 1990 to the beginning of this experiment can be found in Xu et al. (2022).

### Experiment design, soil sampling, and analysis

Almost 10 hetares of harvested sites of eucalypt plantation were divided into four equal blocks, and three planting patterns were randomly arranged within each block in July 2016. The first planting pattern (E) was the continuous planting of pure *Eucalyptus. urograndis* (hybrid strain of *Eucalyptus urophylla* and *Eucalyptus grandis*) plantations of the third generation at a density of 1,667 plants/ha. The second planting pattern (EC) was the creation of mixed plantations of *E. urograndis* and *Cinnamomum camphora* (mixed pattern: inter-row, mixed density: 1667 plants/ha). The third planting pattern (EH) was the creation of mixed plantations of *E. urograndis* and *Castanopsis hystrix* (mixed pattern: inter-row, mixed density: 1667 plants/ha). Simultaneously, four unmanaged first-generation *E. urophylla* plantations in Luogangling Forest Park were selected as controls (CK). Information on forestland preparation, seedling specifications of eucalypts and native trees, and later plantation tending can be found in Xu et al. (2022).

Sixteen mixed topsoil samples in the 10-cm layer were collected in December 2019 by removing the humus and litterfall from four different planting patterns. Soil samples for fungal community structure analysis were preserved with dry ice in centrifuge tubes and transferred to a-80°C freezer as soon as possible. Other soil samples for analyses of soil chemical properties and enzyme activities were stored in a portable refrigerator at 4°C.

The pH of each sample was determined with an electronic pH meter (soil: water, 1:2.5). Soil OM was determined by the potassium dichromate-sulfate colorimetric method (Sims and Haby, 1971). Total nitrogen (TN) and total phosphorus (TP) were measured with the Kjeldahl method (Tsiknia et al., 2014) and sodium hydroxide fusion-molybdenum antimony colorimetric method (Liu H. et al., 2017), respectively. Nitrate nitrogen (NO¯ 3_N) was determined by 2 mol·L^−1^ KCl leaching-indophenol blue colorimetric method and ammonium nitrogen (NH+ 4_N) was determined by UV spectrophotometry (Lu, 1999). Available phosphorus (AP) was measured by the hydrochloric acid-ammonium fluoride extraction-molybdenum antimony colorimetric method (Lu, 1999). Soil available zinc (AZn) and available calcium (ACa) were measured by hydrochloric acid extract, atomic absorption spectrophotometry and ammonium acetate exchange, atomic absorption spectrophotometry, respectively (Liu J. et al., 2017). For soil enzyme activities, acid phosphatase (ACP) was determined by Phenylphosphonium-4-amino-antipyrine colorimetric method (Guan, 1986), urease (URE) by alkaline dish diffusion-HCL titration method (Guan, 1986), and invertase (INV) by 3,5-Dinitrosalicylic acid colorimetric method (Lu, 1999).

### Fungal DNA amplicon sequencing, data processing, and qPCR

Total DNA of the soil microbial community was extracted using an E.Z.N.A.^®^ soil DNA kit (Omega Bio-tek, Norcross, GA, United States) according to the manufacturer’s instructions. The quality of DNA extraction was determined by 1% agarose gel electrophoresis, and DNA concentration and purity were measured using NanoDrop2000 (Thermo Scientific, Wilmington, DE, United States). To amplify the fungal ITS region, the primer pair ITS1F (5′-CTTGGTCATTTAGAGGAAGTAA-3′) and ITS2R (5′-GCTGCGTTCTTCATCGATGC-3′) was used. PCR amplification was performed using ABI GeneAmp^®^ 9,700 and the products were purified using an AxyPrep DNA Gel Extraction Kit (Axygen Biosciences, Union City, CA, United States). Subsequently, the recovered products were detected by 2% agarose gel electrophoresis and quantified using a Quantus™ Fluorometer (Thermo Fisher Scientific Inc., Waltham, MA, United States). After library construction using the NEXTFLEX Rapid DNA-Seq Kit (BiooScientific, Texas, United States), sequencing was performed using Illumina’s Miseq PE300 platform.

The raw 16S rRNA gene sequencing reads were quality-filtered by Trimmomatic and merged by FLASH with the following criteria: (i) the 300 bp reads were truncated at any site receiving an average quality score of <20 over a 50 bp sliding window, and the truncated reads shorter than 50 bp were discarded, reads containing ambiguous characters were also discarded, (ii) only overlapping sequences longer than 10 bp were assembled according to their overlapped sequence. The maximum mismatch ratio of overlap region is 0.2. Reads that could not be assembled were discarded, and (iii) Samples were distinguished based on the barcode and primers, and the sequence direction was adjusted, exact barcode matching, 2 nucleotide mismatch in primer matching. All sample sequences were clustered into different operational taxonomic units (OTUs) based on the similarity between sequences at the 97% similarity level with UPARSE version 7.1 (Edgar, 2013), and chimeric sequences were identified and removed. A representative sequence of each OTU representative sequence was analyzed by RDP Classifier^1^ against the SILVA database (v132) (Zhou et al., 2022). To reduce the impact of sequencing depth on subsequent community composition analysis, the sequence number of each sample was refined to 47,310 sequences. The raw reads were deposited into the NCBI Sequence Read Archive (SRA) database (PRJNA918824).

Mothur software (v1.30.2) was invoked to calculate the Shannon-Wiener, Chao1, and Shannoneven index values for each sample. Real-time quantitative PCR (qPCR) was performed to determine the copy number of fungal ITS genes by using the primer pair ITS1F and ITS2R. For a detailed process of qPCR, refer to Xu et al. (2021b). The nutritional types and functional groups (functional guilds) of the fungal communities were initially assigned by running the “FUNGuild” algorithm,^2^ including “highly probable,” “probable,” and “possible” confidence levels. To avoid over-interpretation of the functional guild, only the “highly probable” and “probable” confidence levels were retained (Nguyen et al., 2016).

### Statistical analysis

The soil chemical properties, enzyme activities, fungal ITS gene copies, alpha diversities, network topology coefficients, and microbial community composition and functional guild were examined by one-way analysis of variance (ANOVA), with Tukey’s honestly significant difference (HSD) test to determine the significant differences among treatments. Non-metric multidimensional scaling (NMDS) analysis was used to visualize the overall dissimilarities in fungal communities among silvicultural treatments, and PERMANOVA (Adonis) was performed to verify the significance of differences in community structure. Mantel tests were applied to study the effect of soil abiotic and biotic information on the microbial community structure, diversity, and functional composition. Redundancy analysis was used to determine whether soil properties were correlated with fungal phylum and functional guild under different woodland treatments (Li et al., 2022).

To further explore fungal species (based on OTU levels) interactions, co-occurrence networks based on Spearman’s correlation matrices of fungal OTUs were constructed using the WGCNA R package (Xu et al., 2021a). Only OTUs with a relative abundance greater than 0.01% were included in the analysis, and random matrix theory (RMT) was implemented to use 0.86 as the appropriate similarity threshold for the test area (Luo et al., 2006). The Benjamini–Hochberg procedure was applied to compute the q values to adjust for multiple hypothesis testing and false discovery rate (Benjamini et al., 2006). The topological characteristics of the fungal network for each soil sample were implemented through subgraph functions by using igraph packages (Qiu et al., 2021). Network complexity was reflected by evaluating topological information such as the number of nodes, edges, edge density, degree centralization and betweenness centralization. Network structural stability was reflected by evaluating natural connectivity of subgraph (Xu et al., 2021a). The R v3.6.1 software platform (R Core Team, 2019) was used to perform all statistical analyses and visualize the results.

## Results

### Soil chemical properties and enzyme activities

**Figure 1:**
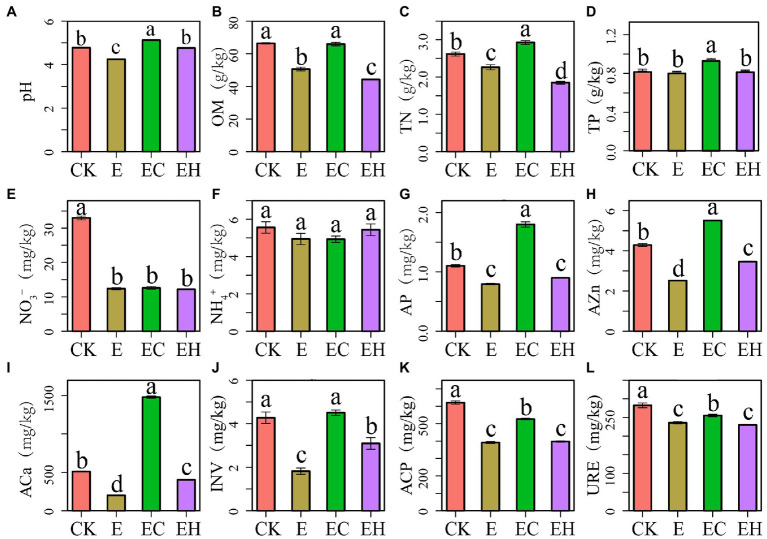
Soil chemical properties **(A–I)** and enzyme activities **(J–L)** in pure eucalypt plantations (CK, E) and mixed plantations (EC, EH). Different lowercase letters above the bar chart in different colors indicate significant differences at the 0.05 level. CK, the first rotation of *E. urophylla* plantations; E, the third rotation of *E. urograndis* plantations; EC, mixed plantations of *E. urograndis* and *Cinnamomum camphora*; EH, *E. urograndis* and *Castanopsis hystrix*; OM, soil organic matter; TN, total nitrogen; TP, total phosphorus; NO¯ 3, nitrate nitrogen; NH+ 4, ammonium nitrogen; AP, available phosphorus; AZn, available zinc; ACa, available calcium; INV, invertase; ACP, acid phosphatase; URE, urease.

Continuous planting management and mixed treatments both induced significant changes in soil chemical properties and enzyme activities (Figure 1). The third-generation eucalypt plantations E had significantly reduced soil pH, OM, TN, NO¯ 3_N, AP, AZn, ACa, INV, ACP, and URE compared with the first-generation eucalypt plantations CK. In contrast, soil pH, almost all nutrient concentrations, and soil enzyme activities increased significantly with the conversion of E to EC. EH also showed significant improvement in soil pH, AZn, ACa, and INV (*p* < 0.05).

### Soil fungal abundance and diversiy

**Figure 2:**
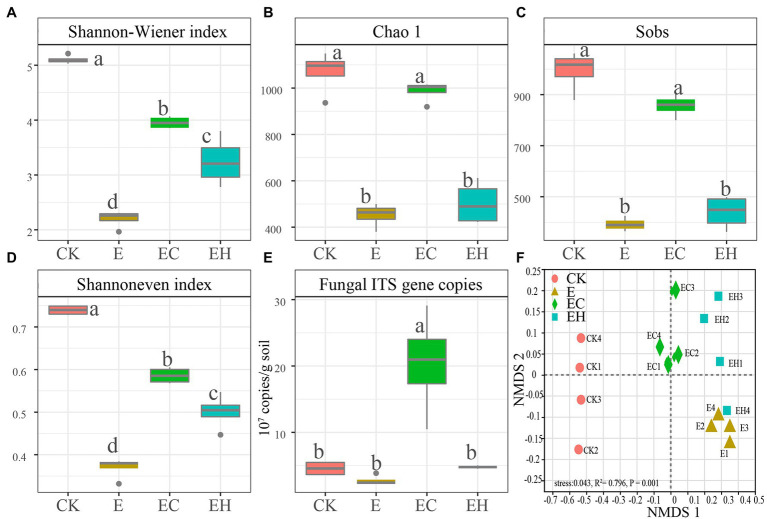
Soil fungal community diversity and gene copies in pure eucalypt plantations (CK, E) and mixed plantations (EC, EH). **(A)** Shannon–Wiener index, **(B)** Chao 1 index, **(C)** Sobs index: number of OTUs actually observed, **(D)** Shannon even index, **(E)** Fungal ITS gene copies. Different lowercase letters above the bar chart in different colors indicate significant differences at the 0.05 level, and **(F)** Non-metric multidimensional scaling analysis of soil fungal communities base on Bray–Curtis distance. CK, the first rotation of *E. urophylla* plantations; E, the third rotation of *E. urograndis* plantations; EC, mixed plantations of *E. urograndis* and *Cinnamomum camphora*; EH, *E. urograndis* and *Castanopsis hystrix*.

After quality control, a total of 1,604,195 optimized sequences were obtained from 16 samples and clustered into 3,194 OTUs, while 33,407 reads per sample were selected for downstream analysis. The Sobs dilution curves constructed from randomly sampled DNA sequences tended to be flat, indicating that most of the fungal diversity had been captured (Supplementary Figure S1). Compared with CK, the Shannon–Winener index, Chao1, Sobs, and Shannoneven indices characterizing soil alpha-diversity and fungal gene copy number of continuous planting pattern E decreased by 56.7, 56.9, 60.5, 49.9 and 39.8%, respectively. However, mixed plantations EC and EH significantly enhanced soil fungal Shannon–Winener and Shannoneven indices compared with E. Meanwhile, EC had a marked advantage in increasing the richness index and fungal gene copy number, and there was no significant difference between EH and E (Figures 2A–E).

### Soil fungal community composition and functional guilds

**Figure 3:**
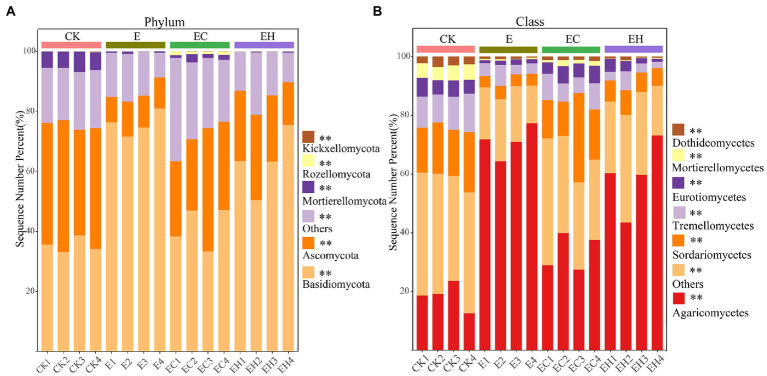
Soil fungal community composition at phylum **(A)** and class **(B)** level in pure eucalypt plantations (CK, E) and mixed plantations (EC, EH). * represents significant differences in the abundance of fungal taxa within different types of plantations. CK, the first rotation of *E. urophylla* plantations; E, the third rotation of *E. urograndis* plantations; EC, mixed plantations of *E. urograndis* and *Cinnamomum camphora*; EH, *E. urograndis* and *Castanopsis hystrix*.

**Figure 4:**
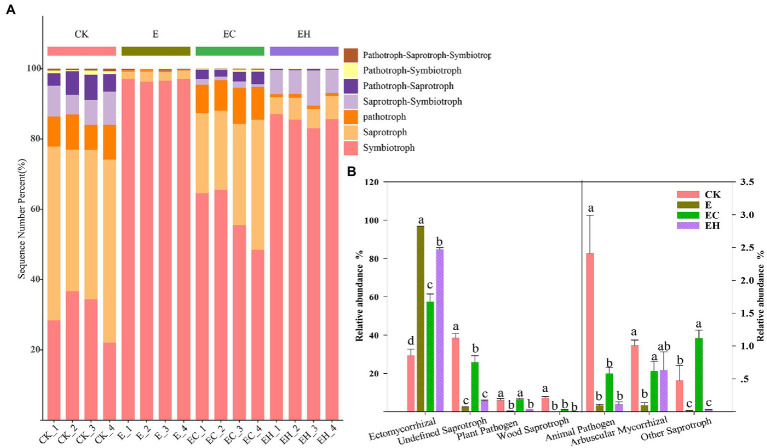
Soil fungal functional information inferred by FUNGuild in pure eucalypt plantations (CK, E) and mixed plantations (EC, EH). **(A)** Major trophic modes of soil fungi and **(B)** variation in the relative abundance of functional guilds (top 7) for each nutrient mode. CK, the first rotation of *E. urophylla* plantations; E, the third rotation of *E. urograndis* plantations; EC, mixed plantations of *E. urograndis* and *Cinnamomum camphora*; EH, *E. urograndis* and *Castanopsis hystrix*.

Continuous planting of pure and mixed planting patterns significantly altered soil fungal community structure beta-diversity (except between E and EH) and relative abundance of fungi at different levels (Figures 2F, 3; Supplementary Table S1, *p* < 0.05). Basidiomycota, Ascomycota, and Mortierellomycota were the three dominant fungal phyla (relative abundance >1%). Compared with CK, the continuous planting pattern E significantly increased the relative abundance of Basidiomycota and decreased the relative abundance of Ascomycota, while the relative abundances of these two dominant fungal phyla in EC and EH were between those of CK and E (Figure 3A; Supplementary Table S2, *p* < 0.05). At the class level, the dominant fungal groups included Agaricomycetes, Sordariomycetes, Tremellomycetes, Eurotiomycetes, Mortierellomycetes, and Dothideomycetes. Among these, planting pattern E caused marked increases in Agaricomycetes and decrease in Sordariomycetes, Eurotiomycetes, Dothideomycetes, Tremellomycetes, and Mortierellomycetes compared with CK. In contrast, EC significantly reduced the relative abundance of Agaricomycetes and increased the abundance of other dominant fungi at the class level compared with E. However, EH did not differ significantly from E in the relative abundances of Sordariomycetes, Eurotiomycetes, Mortierellomycetes, and Dothideomycetes (Figure 3B; Supplementary Table S3, *p* < 0.05). Different plantation practices also induced significant changes in fungal trophic modes (Figure 4, *p* < 0.05). Most of the fungi were classified as symbiotrophs (67.75%), saprotrophs (20.54%), and pathotrophs (4.85%) (Supplementary Table S4). Continuous planting pattern E significantly increased the abundance of symbiotrophs, mainly ECM fungi, while AM fungi showed the opposite. Additionally, E decreased the abundance of saprotrophs, including undefined saprotrophs, wood saprotrophs, and other saprotrophs, and pathotrophs, such as plant pathotrophs and animal pathotrophs. Although the abundance of symbiotrophs and saprotrophs in EC and EH were between those of CK and E, most of the functional guilds did not differ greatly among E and EH (Figure 4). Furthermore, the relative distribution of the functional guilds was similar to the results of NMDS analysis and soil nutrient status in the different plantations (Figures 1, 2f, 4).

### Soil fungal network complexity

**Figure 5:**
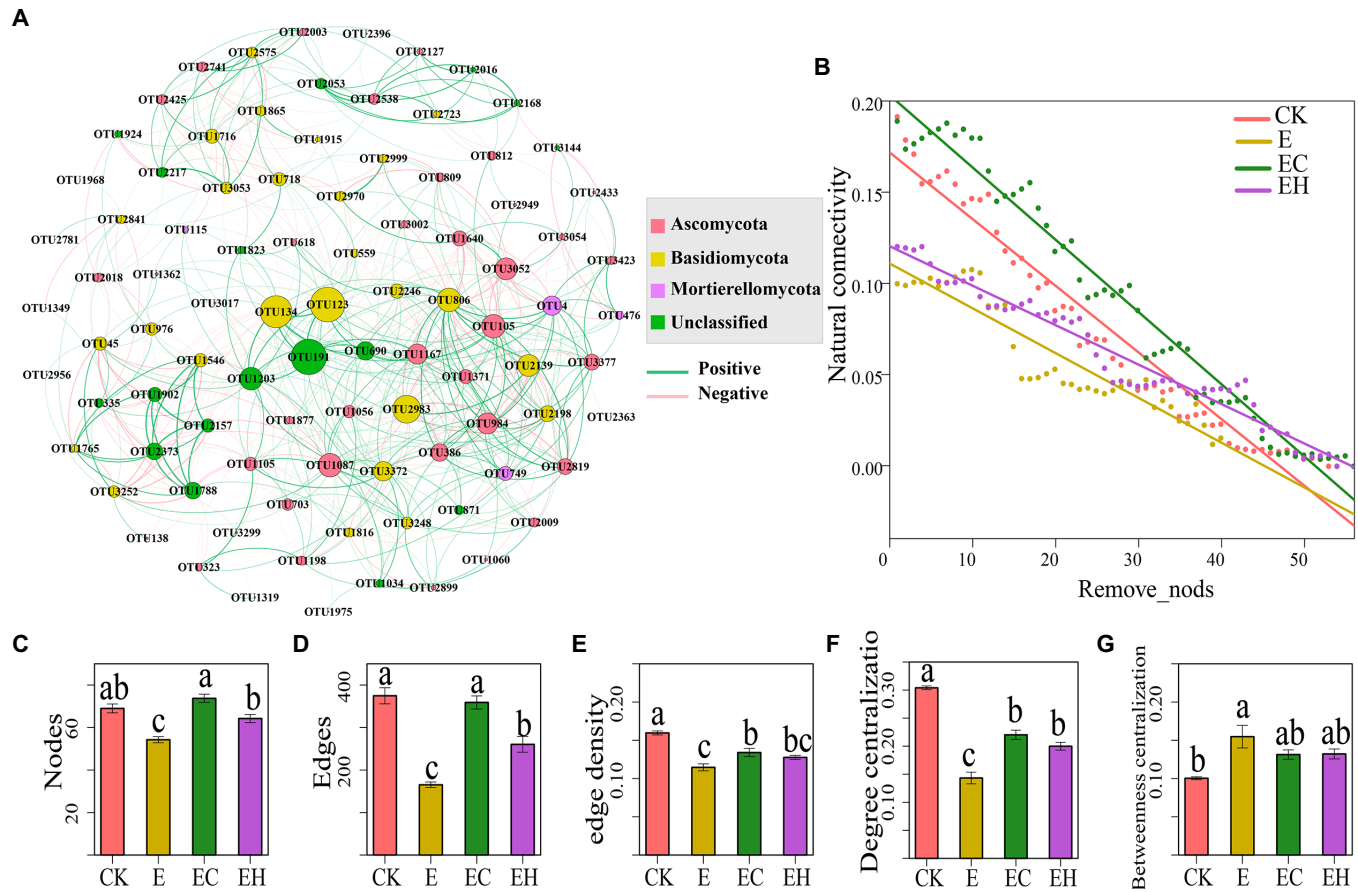
Co-occurrence network and topological characteristics of soil fungal communities in pure eucalypt plantations (CK, E) and mixed plantations (EC, EH). **(A)** Soil fungal co-occurrence network. The Green and pink lines represent positive and negative correlations between OTUs, respectively, **(B)** Natural connectivity of soil microbial networks in relation to the number of removal nodes, **(C)** Nodes: node number, **(D)** Edges: edge number, **(E)** Edge density, **(F)** Degree centralization, **(G)** Betweenness centralization Complexity. CK, the first rotation of *E. urophylla* plantations; E, the third rotation of *E. urograndis* plantations; EC, mixed plantations of *E. urograndis* and *Cinnamomum camphora*; EH, *E. urograndis* and *Castanopsis hystrix*. Different lowercase letters above the bar chart in different colors indicate significant differences at the 0.05 level. The error bars are the standard errors of the mean values of the network topology parameters. The network topological characteristics of each sample were extracted in subgraph function.

Soil fungal network topographic information varied significantly in plantations with different management practices (Figure 5; Supplementary Figure S2, *p* < 0.05). Compared with CK, the network nodes, edges, edge density, and degree centralization index of planting pattern E decreased by 26.5, 57.6, 21.2, and 60.55%, respectively, while betweenness centralization increased by 42.3% (Supplementary Table S5). E also decreased the proportion of positive correlations among fungi. However, network nodes, edges, edge density, degree centralization, and positive correlations were elevated after changing from pure to mixed silviculture, and these indicators were significantly enhanced in EC (Figures 5C–G; Supplementary Table S6). Moreover, with the removal of nodes, the natural connectivity of the fungal network in EC and EH was considerably higher than that in E, indicating that the mixed planting pattern markedly improved the robustness of the fungal network (Figure 5B).

### Relationships between soil chemical properties, enzymes, and fungal community characteristics

**Figure 6:**
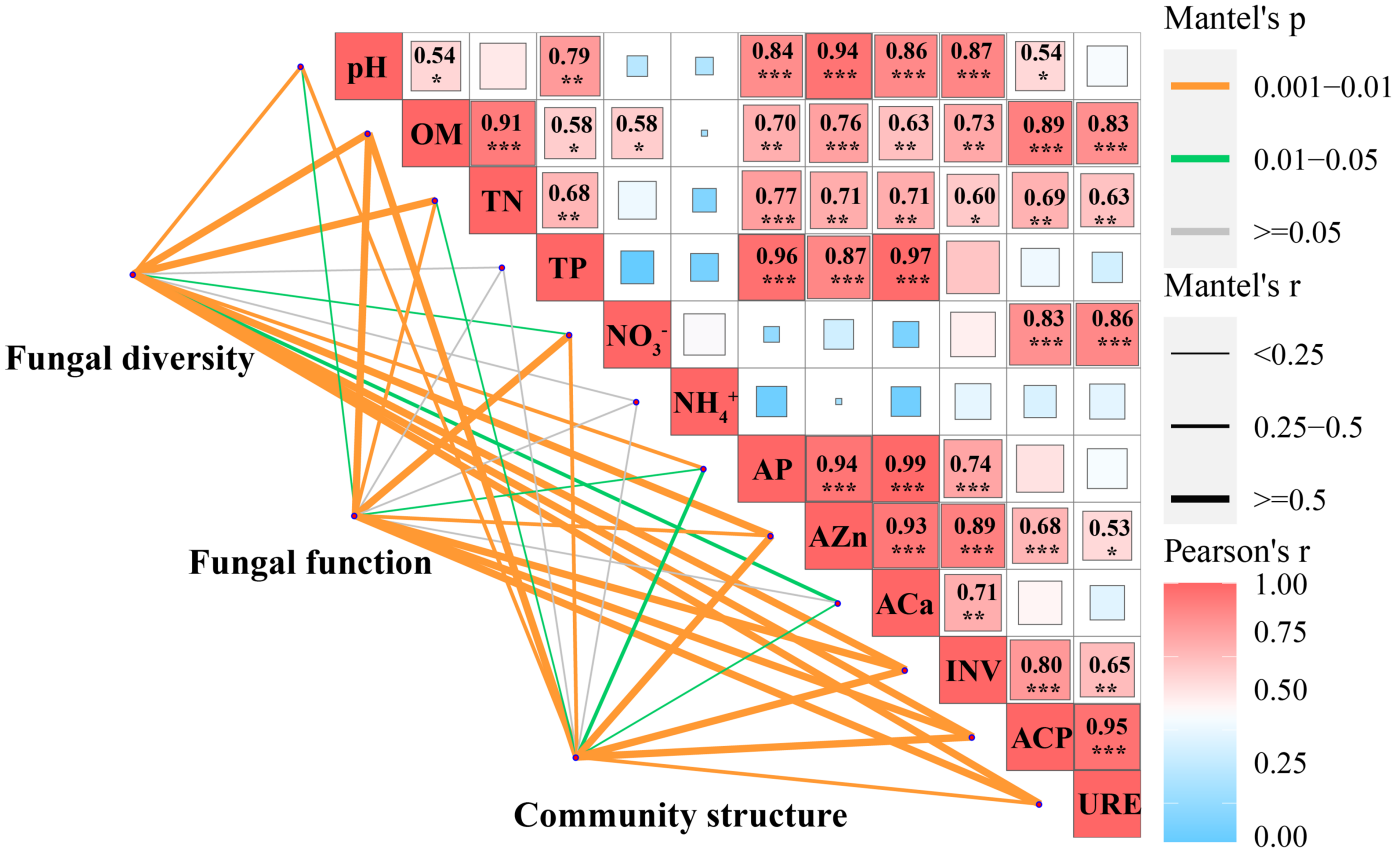
Soil biotic and abiotic factors influencing the fungal community diversity, structure, and function in pure eucalypt plantations (CK, E) and mixed plantations (EC, EH). CK, the first rotation of *E. urophylla* plantations; E, the third rotation of *E. urograndis* plantations; EC, mixed plantations of *E. urograndis* and *Cinnamomum camphora*; EH, *E. urograndis* and *Castanopsis hystrix*; OM, soil organic matter; TN, total nitrogen; TP, total phosphorus; NO¯ 3, nitrate nitrogen; NH+ 4, ammonium nitrogen; AP, available phosphorus; AZn, available zinc; ACa, available calcium; INV, invertase; ACP, acid phosphatase; URE, urease.

**Figure 7:**
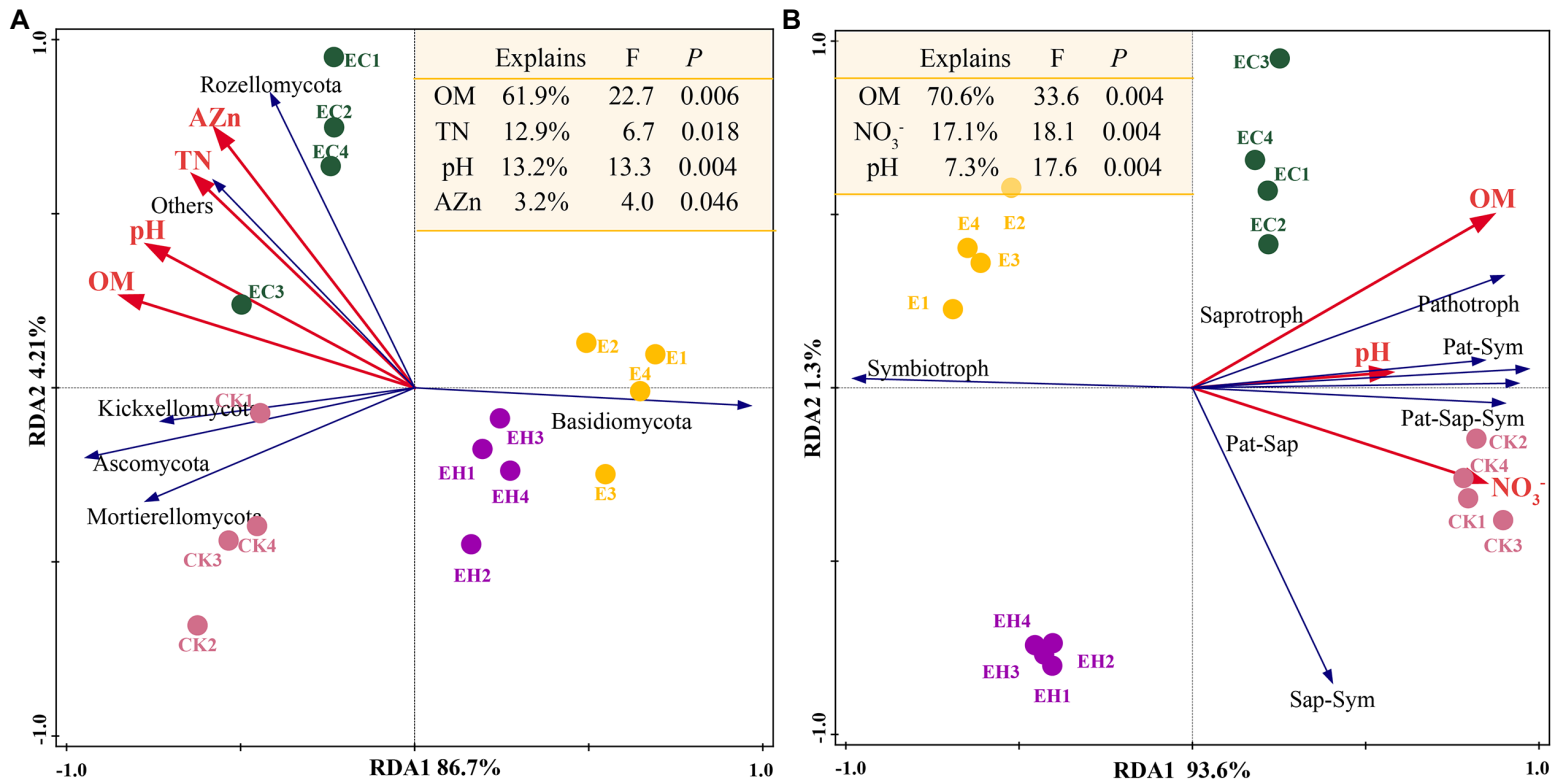
Effect of soil chemistry on fungal community composition and function. Redundancy analysis reveals the relationship between environmental factors and fungal community composition **(A)** or fungal trophic patterns **(B)**. OM, soil organic matter; TN, total nitrogen; TP, total phosphorus; NO¯ 3_N, nitrate nitrogen; NH+ 4_N, ammonium nitrogen; AP, available phosphorus; AZn, available zinc; ACa, available calcium; Sap-Sym, saprotroph-symbiotroph; Pat-Sap, pathotroph-saprotroph; Pat-Sym, pathotroph-symbiotroph; Pat-Sap-Sym, pathotroph-saprotroph-symbiotroph.

Soil fungal diversity, community structure, and functional composition were significantly correlated with soil chemical properties and enzyme activities (Figure 6; Supplementary Figure S3). In particular, fungal community diversity, abundance, and evenness were positively correlated with soil pH, OM, NO¯ 3_N, AZn, INV, ACP, and URE. Furthermore, the dominant fungal groups, such as Ascomycota, Mortierellomycota, and Kickxellomycota, were positively correlated with pH, OM, NO¯ 3_N, INV, ACP, and URE; an exception was Basidiomycota, which showed the opposite pattern and were widely distributed in E (Figures 4, 7). For functional groups, the abundances of saprotrophs and pathotrophs were positively correlated with soil pH, OM, TN, NO¯ 3_N, AZn, INV, ACP, and URE, while symbiotrophs were negatively linked to soil nutrients and enzymatic activities (Figures 6, 7; Supplementary Figure S3). Redundancy analysis revealed that soil OM, TN, pH, and AZn collectively explained 91.2% of the soil fungal community structure variables, and soil OM, NO¯ 3_N, and pH jointly explained 95.0% of the soil functional profile variables. Thus, soil OM and pH together shaped and drove changes in fungal community structure and function in this study.

## Discussion

### Effect of plantation conversion on fungal diversity

Continuous and mixed planting management patterns induced notable changes in fungal abundance and diversity (Figure 2). The third generation of the eucalypt plantations E reduced soil ITS gene abundance as well as fungal diversity, richness, and evenness compared with the control plantation (CK). The transition from E to mixed plantations (EC, EH) caused a significant rebound in soil fungal diversities (except for the EH richness index). This phenomenon was also observed during the transformation of other pure plantations, such as *E. grandis* (Pereira et al., 2021) and *Robinia pseudoacacia* (Dong et al., 2021), and may be attributed to the marked differences in soil chemical properties and enzyme activities caused by different management patterns (Xu et al., 2020). Yang et al. (2019) and Guo et al. (2022) reported that soil fungal diversity and abundance were highly positively correlated with soil pH and organic C and N contents. Similarly, soil pH, OM, NO¯ 3_N, and AZn contents maintained a highly positive relationship with fungal diversity, richness, and evenness in this study. In addition, the multi-generational succession of eucalypt plantations induced a large accumulation of single low-quality litter. As a major source of carbon for the topsoil (Li et al., 2021), this low quality litter negatively affected fungal diversity and abundance (Xu et al., 2021a). However, the multi-layered mixed plantations markedly improved litter quality and increased dead root diversity, providing an adequate source of C and N for fungal growth, as well as a larger root surface area and more diverse habitat (Rottstock et al., 2014).

### Influence of plantation conversion on fungal community composition and functional groups

Different plantation management patterns changed the structural composition of the soil fungal community. The fungi with the top three relative abundances—Basidiomycota, Ascomycota, and Mortierellomycota—are shared by many other forest soils and are regarded as core microorganisms (Rottstock et al., 2014; Xu et al., 2020). Among them, Agaricomycetes belonging to Basidiomycota, commonly defined as oligotrophic fungi, act as common ECM symbionts of forest trees (Liu et al., 2020). Here, E caused a significant increase in the relative abundance of Agaricomycetes, which could be attributed to plant nutrient stress caused by soil nutrient deprivation, thus increasing the dependence of trees on fungal symbionts (Read and Perez-Moreno, 2003). Furthermore, the relative abundances of Tremellomycetes, Sordariomycetes, and Dothideomycetes belonging to the Ascomycota and Eurotiomycetes belonging to Basidiomycota decreased significantly in the third generation of eucalypt plantations (E). However, EC significantly mitigated the changes in abundance of dominant fungi caused by continuous planting (Figure 4; Supplementary Table S3). It was previously reported that Sordariomycetes, Dothideomycetes, and Eurotiomycetes were the major cellulose-and lignin-degrading fungi (Fan et al., 2012; Wilhelm et al., 2017; Wu et al., 2019), while members of Tremellomycetes have a good wood-degrading ability (Wu et al., 2019). Therefore, the differential distribution of these decomposer fungi taxa in different plantations may explain the changes in soil fertility caused by the shift in management patterns.

The shift in plantation management patterns also induced marked differences in fungal functional guilds (Castano et al., 2019). The relative abundance of soil symbiotrophs such as ECM was significantly elevated in the third generation eucalypt plantation E, accompanied by a decrease in soil pH, OM, and nutrient contents, while the relative abundance of saprotrophs and pathotrophs, including soil undefined saprophytes, phytopathogens, wood saprophytes, animal pathogens, and other saprophytes, were significantly decreased compared with those of CK. In contrast, mixed plantation EC significantly reduced the relative abundance of symbiotrophs and elevated the relative abundance of saprotrophs and pathotrophs, but EH not significantly different compare with E (Figure 4). A possible reason for this is that symbiotic fungi, such as ECM, often act as nutrient channels for trees and functional extensions of roots, increasing the surface area for the uptake of soluble nutrients (Lei et al., 2022). In addition, ECM fungi benefit from OM decomposition through increased N mobilization rather than the release of metabolic C, which facilitates the mitigation of soil nutrient decline and plant nutrient stress (Lindahl and Tunlid, 2015). Unlike symbiotic fungi, saprophytes possess a strong decomposition capacity for litter and roots (Yao et al., 2017), with predominant role of maintaining ecosystem nutrient homeostasis (Marty et al., 2019). Recent studies confirmed that soil saprophytic and pathogenic fungal abundances were positively correlated with soil fertility (Kyaschenko et al., 2017), which was also verified in the present study. Meanwhile, Sordariomycetes, Dothideomycetes, and Eurotiomycetes were significantly enriched in mixed plantations in the form of saprophytic or pathogenic fungi.

Soil enzyme activity, as one of the functional manifestations of soil, is notably influenced by plantation management patterns (Guo et al., 2022). Although soil extracellular enzymes are secreted by both bacteria and fungi, their activity is mainly driven by fungi (Jin et al., 2019). Soil fungi produce various extracellular enzymes that are instrumental in the decomposition of refractory litter, regulation of soil nutrient balance, and soil carbon and nitrogen mineralization (Benoit et al., 2015). In this study, INV, URE, and ACP activities related to the regulation of C-, N-, and P-cycles, respectively, were strongly and positively correlated with fungal diversity and the abundance of functional groups (Figure 6), such as saprophytic and pathogenic fungi, and negatively related to symbiotic fungi. This demonstrates the strong ability of fungi to regulate soil ecological functions (Li et al., 2021).

### Responses of fungal community network structure to plantation conversion

Soil quality degradation induces associative reorganization of fungal taxa, which reduces fungal community network complexity and stability, and weakens ecosystem multi-functionality (Luo et al., 2023). This finding was confirmed in the present study, with the third generation eucalypt plantation having a significantly diminished number of nodes, edges, network density, degree centralization, and robustness that characterize the complexity and stability of soil fungal networks. In contrast, the topological metrics related to the complexity and robustness of the soil fungal network were elevated in the mixed plantations, and EC reached significant levels (Figure 5, *p* < 0.05). A possible explanation for these differences may be that soil nutrient deprivation in continuous planting pattern E caused a large number of fungi to become dormant, resulting in the simplification and/or loss of interactions between fungi and a significant negative impact on community complexity and stability (Che et al., 2019; Nakayama et al., 2019). Compared with pure forests, mixed plantations provide sufficient substrate and diverse habitats for fungal colonization, which consequently enhances fungal mycelial connectivity and significantly increases the complexity and stability of microbial networks (Pereira et al., 2021). In the present study, E significantly increased the proportion of negative correlations between fungal OTUs compared with CK, while the proportion of positive correlations between fungi in mixed plantations (EC and EH) was markedly increased. This phenomenon was also found during the transformation of eucalypt pure plantations to eucalypt – acacia mixed plantations (Pereira et al., 2021). It may be attributed to reduced competition from the diverse understory habitat resources provided by mixed forest ecosystems; Thus, they may allow more species to maintain free-living populations (Fan et al., 2018; Zhu et al., 2021). However, there are also studies confirming that negative correlations among microorganisms do not warrant competition between taxa (Van Der Heijden and Hartmann, 2016). Therefore, the reasons for the significant changes in the positive and negative correlations between soil fungi in different plantations need to be further investigated.

### Key soil factors correlated with fungal communities

Mantel tests and RDA analysis showed that fungal diversity, community structure, and functional groups were significantly influenced by soil OM, pH, TN, and NO¯ 3_N together (Figures 6, 7; Supplementary Figure S3). The quality of OM influences the microbial composition (Tscherko et al., 2005), and its species and quantity play critical roles in determining the structure and function of heterotrophic microbial communities (Merilä et al., 2010). This study confirmed that continuous planting of eucalypts (E) decreased soil OM and nutrient contents while increasing the dependence of the root system on symbiotic fungi. The implantation of native tree species in mixed plantations leads to changes in litter composition and quantity, hence the proportion of saprophytic fungi increased significantly in these plantations. Soil pH, as a major influencing factor of microbial structure dynamics, directly shapes fungal communities by affecting enzyme activity and mycorrhizal colonization (Deacon, 2006). In this study, soil fungal diversity, community structure, and OM changes with plantation management patterns are also closely related to soil pH (Guo et al., 2022). Phosphorus is considered a key regulator of fungal community biogeographic patterns, and adequate phosphorus levels had positive effects on mycelial development and mycorrhizal formation (Liu et al., 2022). However, soil fungal communities in the experimental area of the present study were more restricted by N than P, and fungal community diversity, community structure, and functional guilds were greatly influenced by TN and NO-3_N to varying degrees. This may be attributed to the growth stage of eucalypt plantations. Young eucalypt plantations are in a vigorous growth stage and the uptake of soil N may form a stronger competition with microorganisms (Turner and Lambert, 2008). Also, the high C: N ratio of eucalypt litter at this growth stage results in N limitation for microbial growth and reproduction (Xu et al., 2018).

## Conclusion

Fungal community structure and function showed different responses to pure and mixed eucalypt plantations. Multi-generational continuous eucalypt planting caused decreases in soil pH, OM, nutrient contents, and enzyme activities related to C, N, and P cycles, and negatively affected fungal diversity and abundance compared with CK. In addition, Agaricomycetes were significantly enriched in E soils. However, although EH only showed significant improvement in fungal diversity and evenness in a short term compared with E, EC also significantly enhanced the relative abundance of symbiotic and/or saprophytic fungi (e.g., Sordariomycetes, Dothideomycetes, Eurotiomycetes, and Tremellomycetes), as well as soil nutrient status and enzyme activity. Soil microbial co-occurrence network analysis revealed that the complexity and robustness of fungal networks in the third-generation pure plantations were significantly lower than those in the mixed plantations. This further confirmed the negative impact of the multi-generational continuous planting pattern on the soil. Therefore, in response to the global trend of conversion of natural forests to plantations, we recommend that suitable tree species are selected to establish mixed forests, such as *E. urograndis* and *Cinnamomum camphora,* to ensure the long-term sustainability of fast-growing plantations.

## Data availability statement

The original contributions presented in the study are publicly available. All the fungal raw sequences have been deposited into the NCBI Sequence Read Archive (SRA) database, accession number PRJNA918824.

## Author contributions

CL: wrote original draft. YX and AD: conceived and designed this study. ZW and WZ: collected soil samples in the plantations and processed samples in the laboratory. All authors contributed to the article and approved the submitted version.

## Funding

This work was financially supported by the Special funds for the basic research and development program in the central non-profit research institutes of China (CAFYBB2022MA006), Guangdong Forestry SciTech Innovation Project (2018KJCX014), Forestry Ecological Monitoring Network Platform Construction Project (2022CG644), and the Operation Project for Guangdong Zhanjiang Eucalyptus Forest Ecosystem National Positioning Observation and Research Station.

## Conflict of interest

The authors declare that the research was conducted in the absence of any commercial or financial relationships that could be construed as a potential conflict of interest.

## Publisher’s note

All claims expressed in this article are solely those of the authors and do not necessarily represent those of their affiliated organizations, or those of the publisher, the editors and the reviewers. Any product that may be evaluated in this article, or claim that may be made by its manufacturer, is not guaranteed or endorsed by the publisher.

## Footnotes

^1^ http://rdp.cme.msu.edu/

^2^ http://www.funguild.org/
