# Supplementary material for: Mixing planting with native tree species reshapes soil fungal community diversity and structure in multi-generational eucalypt plantations in southern China
Source: Front Microbiol. 2023 Feb 22;14:1132875. doi: 10.3389/fmicb.2023.1132875 (PMC9994620; doi:10.3389/fmicb.2023.1132875)
Supplement: Supplementary file 1 [file Data_Sheet_1.docx]

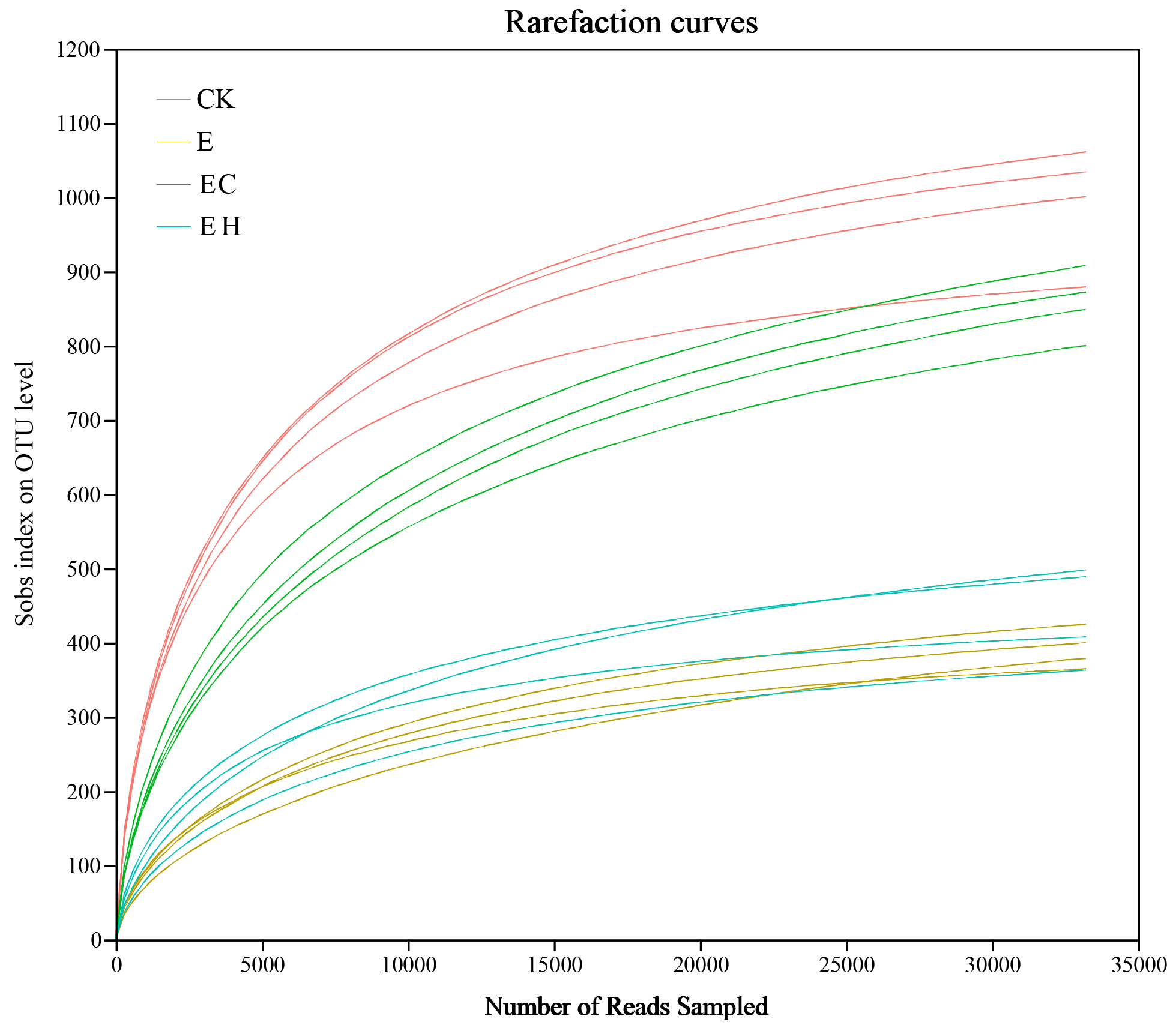


FIGURE S1 The Sobs dilution curves constructed from randomly sampled DNA sequences. CK: the first rotation of *E. urograndis* plantations; E: the third rotation of *E. urograndis* plantations; EC: mixed plantations of *E. urograndis* and *C. camphora*; EH: *E. urograndis* and *C. hystrix.*


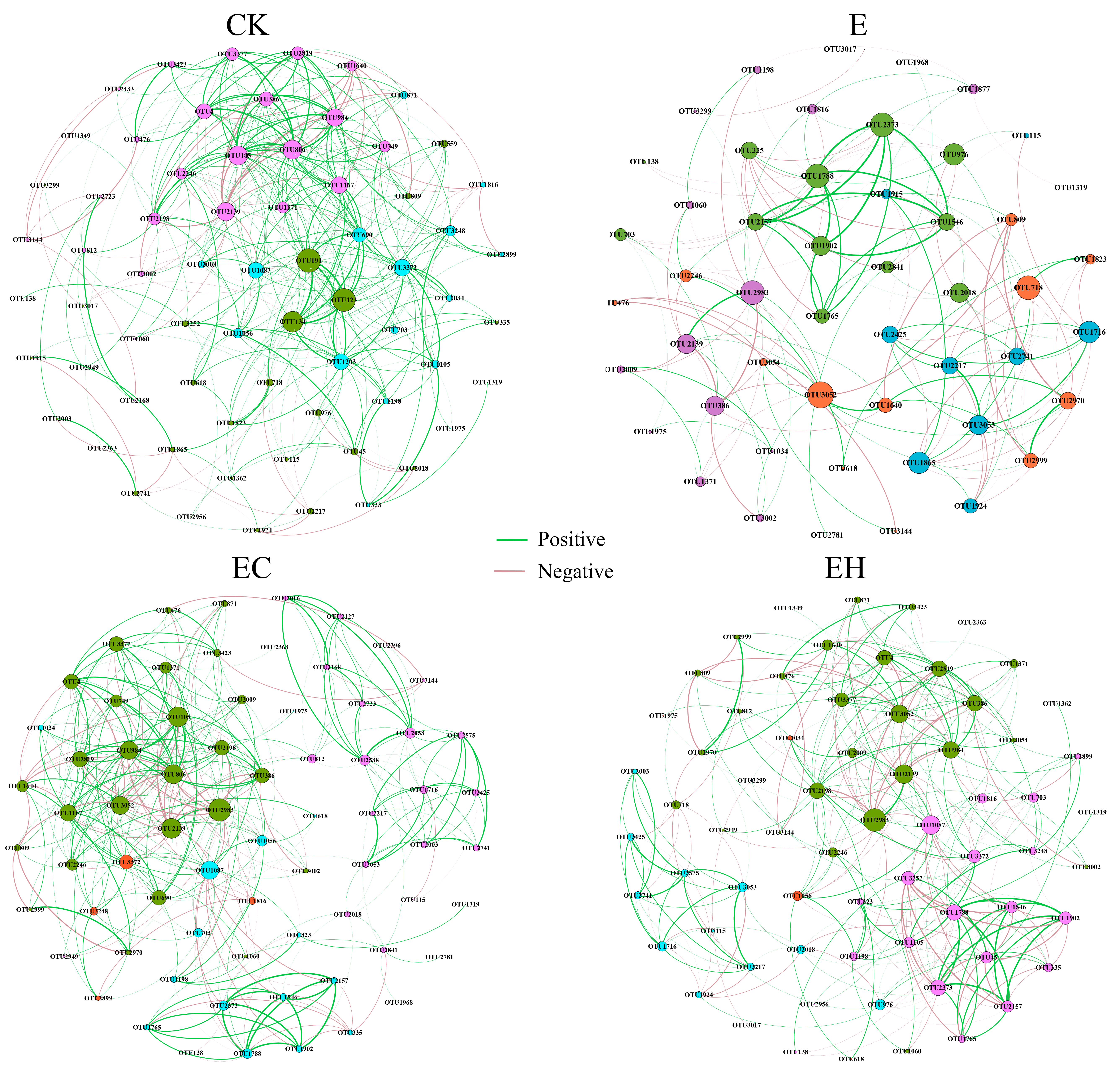


FIGURE S2 Co-occurrence network and topological characteristics of soil fungal communities in plantations CK, E, EC, and EH. CK: the first rotation of *E. urograndis* plantations; CK: the first rotation of *E. urograndis* plantations; E: the third rotation of *E. urograndis* plantations; EC: mixed plantations of *E. urograndis* and *Cinnamomum camphora*; EH: *E. urograndis* and *Castanopsis hystrix*; The Green and pink lines represent positive and negative correlations between OTUs, respectively. The circle pattern represents the different OTUs, the OTUs with the same colour are in the same module; the size of the circle represents the degree, the larger the radius, the greater the degree of the OTU.


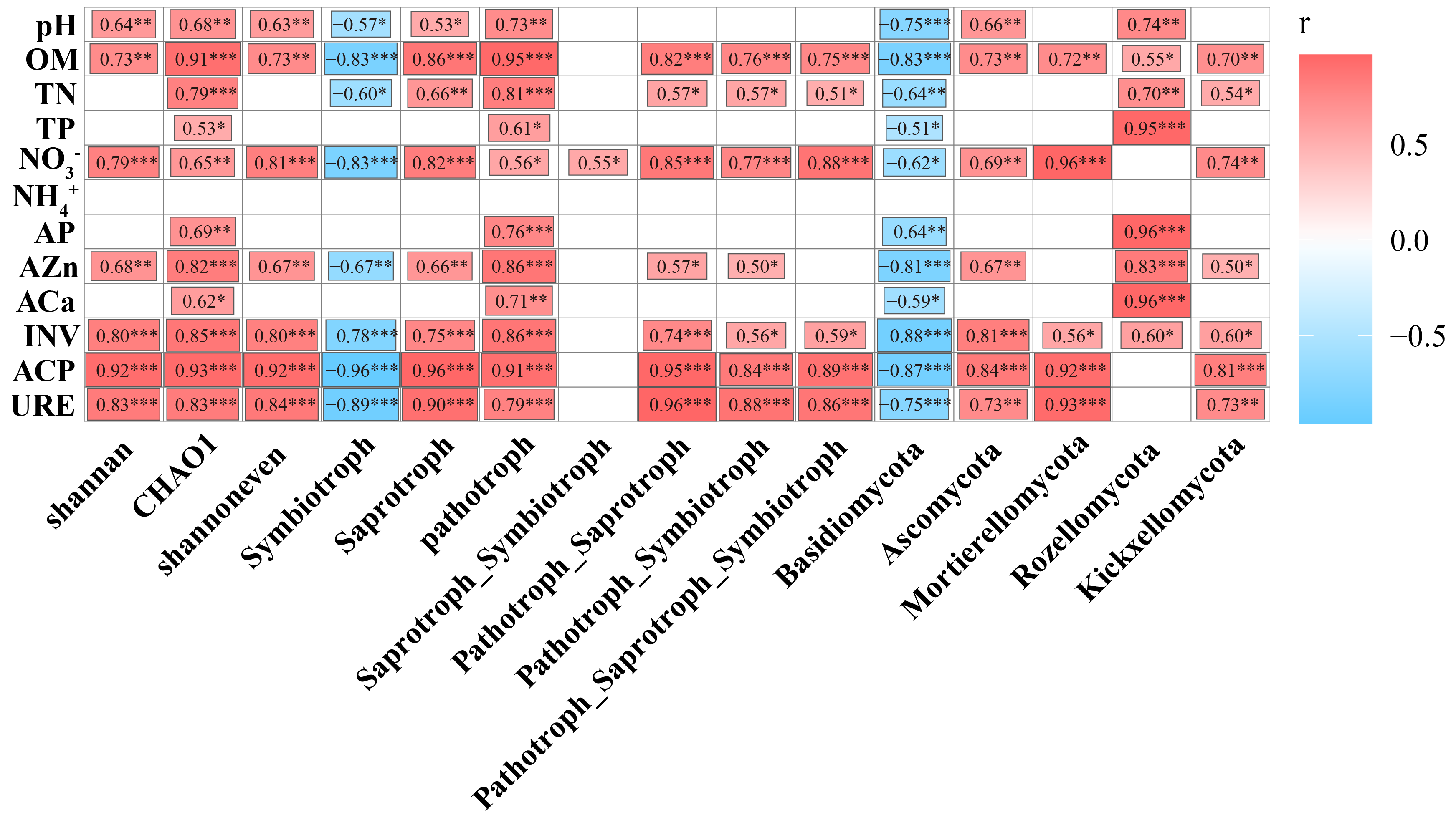


FIGURE S3 Heatmap reflecting the relationship between soil environmental factors and fungal diversities, functional guilds, and relative abundance of dominant fungi at phylum level. Positive and negative correlations are indicated by red and blue, respectively (* *p* < 0.05; ** *p* < 0.01; *** *p* < 0.001).

TABLE S1 Pairwise comparison of soil fungal community structure between CK, E, EC, and EH in the PERMANOVA analysis.

|  | E | EC | EH |
| --- | --- | --- | --- |
| CK | **0.034*** | **0.049*** | **0.037*** |
| E |  | **0.026*** | 0.064 |
| EC |  |  | **0.020*** |

TABLE S2 The relative abundance of top three dominant fungal phyla in pure eucalypt plantations (CK, E) and mixed plantations (EC, EH).

| Taxonomy | Basidiomycota | Ascomycota | Mortierellomycota |
| --- | --- | --- | --- |
| CK | 35.38% ±1.2 c | 39.98% ±1.82 a | 5.85% ±0.31 a |
| E | 75.87% ±1.96 a | 10.23 ±0.67 c | 0.04% ±0.13 c |
| EC | 41.40% ±5.11 c | 29.88% ±3.93 b | 1.63% ±0.38 b |
| EH | 63.14% ±4.46 b | 22.03% ±2.95 b | 0.02% ±0.06 c |

TABLE S3 The relative abundance of top six dominant fungal classes in pure eucalypt plantations (CK, E) and mixed plantations (EC, EH).

| Taxonomy | Agaricomycetes | Tremellomycetes | Sordariomycetes | Dothideomycetes | Eurotiomycetes | Mortierellomycetes |
| --- | --- | --- | --- | --- | --- | --- |
| CK | 18.50% ±2.26 d | 11.09% ±0.74 a | 17.28% ±1.21 a | 2.84% ±0.29 a | 5.38% ±0.38 a | 4.98% ±0.17 a |
| E | 71.10% ±2.68 a | 4.56 ±0.89 c | 4.13% ±0.13 b | 0.72% ±0.11 bc | 1.41 ±0.18 b | 0.40% ±0.08 c |
| EC | 33.47% ±3.10 c | 7.34% ±0.96 b | 18.13% ±4.29 a | 1.22% ±0.08 b | 5.10% ±0.49 a | 1.43% ±0.28 b |
| EH | 59.18% ±6.09 b | 3.67% ±0.95 d | 7.09% ±0.52 b | 0.65% ±0.17 c | 2.68% ±0.79 b | 0.21% ±0.06 c |

TABLE S4 Soil fungal functional guilds information inferred by FUNGuild in pure eucalypt plantations (CK, E) and mixed plantations (EC, EH).

| Taxonomy | CK1 | CK2 | CK3 | CK4 | E1 | E2 | E3 | E4 | EC1 | EC2 | EC3 | EC4 | EH1 | EH2 | EH3 | EH4 |
| --- | --- | --- | --- | --- | --- | --- | --- | --- | --- | --- | --- | --- | --- | --- | --- | --- |
| pathotroph | 8.60% | 10.03% | 7.08% | 9.96% | 0.49% | 0.46% | 0.47% | 0.35% | 8.07% | 8.58% | 10.32% | 9.36% | 0.80% | 1.11% | 1.00% | 0.84% |
| Saprotroph | 49.45% | 40.32% | 42.59% | 52.04% | 2.15% | 2.87% | 2.44% | 2.39% | 22.77% | 22.65% | 28.83% | 37.00% | 4.81% | 6.21% | 5.49% | 6.60% |
| Symbiotroph | 28.39% | 36.66% | 34.38% | 22.07% | 97.02% | 96.36% | 96.57% | 97.08% | 64.61% | 65.47% | 55.50% | 48.46% | 87.12% | 85.49% | 83.11% | 85.64% |
| Pathotroph-Saprotroph | 3.52% | 6.67% | 7.19% | 4.96% | 0.27% | 0.13% | 0.22% | 0.13% | 2.70% | 1.97% | 2.65% | 3.51% | 0.34% | 0.30% | 0.44% | 0.25% |
| Pathotroph-Saprotroph-Symbiotroph | 0.54% | 0.38% | 0.48% | 0.71% | 0.00% | 0.01% | 0.01% | 0.01% | 0.04% | 0.13% | 0.26% | 0.31% | 0.01% | 0.02% | 0.02% | 0.01% |
| Pathotroph-Symbiotroph | 0.78% | 0.34% | 1.25% | 0.88% | 0.04% | 0.14% | 0.21% | 0.03% | 0.25% | 0.22% | 0.67% | 0.53% | 0.03% | 0.09% | 0.03% | 0.02% |
| Saprotroph-Symbiotroph | 8.72% | 5.59% | 7.04% | 9.39% | 0.02% | 0.04% | 0.08% | 0.02% | 1.56% | 0.98% | 1.77% | 0.83% | 6.88% | 6.78% | 9.99% | 6.64% |

TABLE S5 Soil fungal network topographic information in pure eucalypt plantations (CK, E) and mixed plantations (EC, EH).

| Trement | Nodes | Edges | Edge_density | Degree_centralization | Betweenness_centralization |
| --- | --- | --- | --- | --- | --- |
| CK | 68 | 361 | 0.158472 | 0.304214 | 0.095422 |
| CK | 70 | 397 | 0.164389 | 0.299379 | 0.102047 |
| CK | 74 | 413 | 0.152906 | 0.299148 | 0.104356 |
| CK | 64 | 328 | 0.162698 | 0.313492 | 0.099625 |
| E | 50 | 153 | 0.124898 | 0.12 | 0.135788 |
| E | 55 | 162 | 0.109091 | 0.168687 | 0.111533 |
| E | 56 | 162 | 0.105195 | 0.149351 | 0.13437 |
| E | 56 | 184 | 0.119481 | 0.135065 | 0.19735 |
| EC | 70 | 362 | 0.149896 | 0.241408 | 0.173568 |
| EC | 76 | 366 | 0.128421 | 0.218246 | 0.129345 |
| EC | 78 | 391 | 0.130203 | 0.220446 | 0.123145 |
| EC | 71 | 317 | 0.127565 | 0.201006 | 0.149294 |
| EH | 67 | 295 | 0.133424 | 0.215061 | 0.123014 |
| EH | 68 | 286 | 0.125549 | 0.202809 | 0.15116 |
| EH | 60 | 215 | 0.121469 | 0.200565 | 0.172457 |
| EH | 62 | 246 | 0.13009 | 0.181386 | 0.129852 |

TABLE S6 Proportion of positive and negative correlations in the co-occurrence network of soil fungi in pure eucalypt plantations (CK, E) and mixed plantations (EC, EH).

| Taxonomy | Total edges | Positive correlations | Negative correlations |
| --- | --- | --- | --- |
| CK | 375 ±19.00 a | 78.10% ±2.94 a | 21.90% ±2.94 d |
| E | 165 ± 6.60 c | 52.79% ±0.43 d | 47.21% ±0.43 a |
| EC | 359 ±15.40 a | 67.71% ±0.82 b | 32.29% ±0.82 c |
| EH | 261 ±18.53 b | 57.71% ±0.38 c | 42.29% ±0.38 b |
